# Comprehension of own and other species’ alarm calls in sooty mangabey vocal development

**DOI:** 10.1007/s00265-023-03318-6

**Published:** 2023-05-23

**Authors:** Julián León, Constance Thiriau, Catherine Crockford, Klaus Zuberbühler

**Affiliations:** 1 Institute of Biology, University of Neuchâtel, 2000 Neuchâtel, Switzerland; 2 Taï Monkey Project, Centre Suisse de Recherches Scientifiques, 01 BP1303 Abidjan 01, Côte d’Ivoire; 3 Tai Chimpanzee Project, Centre Suisse de Recherches Scientifiques, 01 BP1303 Abidjan 01, Côte d’Ivoire; 4 Institute of Cognitive Sciences Marc Jeannerod, CNRS, 69330 Lyon, France; 5 School of Psychology and Neuroscience, University of St. Andrews, Saint Andrews, KY16 9JP Scotland UK

**Keywords:** Vocal communication, Primates vocalizations, Heterospecific communication, Predation, Alarm calls, Playback experiments

## Abstract

**Abstract:**

Primates understand the meaning of their own and other species’ alarm calls, but little is known about how they acquire such knowledge. Here, we combined direct behavioural observations with playback experiments to investigate two key processes underlying vocal development: comprehension and usage. Especifically, we studied the development of con- and heterospecific alarm call recognition in free-ranging sooty mangabeys, *Cercocebus atys*, across three age groups: young juveniles (1–2y), old juveniles (3–4y) and adults (> 5y). We observed that, during natural predator encounters, juveniles alarm called to a significantly wider range of species than adults, with evidence of refinement during the first four years of life. In the experiments, we exposed subjects to leopard, eagle and snake alarm calls given by other group members or sympatric Diana monkeys. We found that young juveniles’ locomotor and vocal responses were least appropriate and that they engaged in more social referencing (look at adults when hearing an alarm call) than older individuals, suggesting that vocal competence is obtained via social learning. In conclusion, our results suggest that alarm call comprehension is socially learned during the juvenile stage, with comprehension preceding appropriate usage but no difference between learning their own or other species’ alarm calls.

**Significance statement:**

Under natural conditions, animals do not just interact with members of their own species, but usually operate in a network of associated species. However, ontogenetic research on primate communication frequently ignores this significant element. We studied the development of con- and heterospecific alarm call recognition in wild sooty mangabeys. We found that communicative competence was acquired during the juvenile stages, with alarm call comprehension learning preceding appropriate vocal usage and with no clear difference in learning of con- and heterospecific signals. We also found that, during early stages of life, social referencing, a proactive form of social learning, was key in the acquisition of competent alarm call behaviour. Our results show that primates equally learn to interpret alarm calls from their own and other species during their early stages of life and that this learning process is refined as the animals mature.

**Supplementary Information:**

The online version contains supplementary material available at 10.1007/s00265-023-03318-6.

## Introduction

Research in primate communication continues to contribute to theories of speech and language evolution, with growing evidence of a continuum of abilities between human and non-human primates, especially in terms of comprehension (Stensland et al. 2003; Zuberbühler 2003a, b; Slocombe and Zuberbühler 2005; Liebal et al. 2014; Watson et al. 2015; Crockford et al. 2017; Fischer 2017, 2021; Fischer and Price 2017; Quam et al. 2017; Bergman et al. 2019; Ghazanfar et al. 2019). Yet, key differences remain, particularly in terms of vocal production, which is surprisingly limited in non-human primates, but also in vocal usage, with many utterances fixed to specific biological functions (Cheney and Seyfarth 2018; see Hammerschmidt and Fischer 2008 for review). Some flexibility in production and usage has been reported in cases when single vocal units are combined into more complex structures, a relatively novel line of research with considerable potential (see Girard-Buttoz et al. 2022).

Alarm calls have traditionally played a key role in studies of flexibility in vocal production, usage and comprehension (Zuberbühler 2007). Pioneering work on vervet monkey (*Cercopithecus aethiops*) alarm calls has originally suggested human-like semantic abilities (Seyfarth et al. 1980a, b), although more recent work has challenged this view (Wheeler and Fischer 2012; Price et al. 2015). Vervet monkeys produce acoustically distinct alarm calls in response to their key predators (leopards, eagles, snakes) and show appropriate antipredator responses when hearing these calls, e.g., rapidly climbing into trees when hearing leopard alarms versus scanning the sky or running for cover when hearing eagle alarms (Seyfarth et al. 1980a). Follow-up research has produced comparable evidence in other primates, suggesting that such abilities are a general feature of primate cognition and, most likely, many other groups of animals (Fischer and Hammerschmidt 2001; Fischer et al. 2001; Manser 2001; Manser et al. 2001; Fichtel and Kappeler 2002, 2011; Zuberbühler 2003a; Arnold and Zuberbühler 2006; Kirchhof and Hammerschmidt 2006; Townsend and Manser 2013; Fichtel 2020;).

How do primates learn the meaning of their calls? The literature on acquisition and development of non-human primate communication is comparably limited, mainly because it is difficult to continuously monitor animals under field conditions with development likely to be affected by key life experiences (Hauser 1989; Elowson et al. 1992; Owren et al. 1993; Egnor and Hauser 2004; Chow et al. 2015). Classic research on vervet monkeys suggests that full competence in vocal comprehension, usage and production occurs during the first four years of life (Seyfarth and Cheney 1986), albeit with differences in flexibility (Seyfarth and Cheney 2010; Wegdell et al. 2019). One remarkable finding is that infant vervet monkeys give alarm calls in response to a much wider array of species than adults, although in non-arbitrary ways: leopard alarms are produced to a wide range of terrestrial mammals, eagle alarms to many flying objects (including falling leaves) and snake alarms to any snake-like objects and reptiles (Seyfarth and Cheney 1980). Over the course of their development, youngsters then appear to reduce and refine their calling behaviour to the relevant predator species. Whether infants are born with core knowledge of predator classes (e.g., aerial vs terrestrial) or whether they simply observe others and first overgeneralise is currently unknown. It also remains unclear whether communicative competence in the main domains (production, usage, comprehension) emerges either gradually over multiple experiences or suddenly in response to one or a few key experiences.

Regarding comprehension and subsequent use, there is good experimental evidence that learning can be rapid requiring only a small number of experiences. For example, when exposed to a remotely operated drone, an unfamiliar potential aerial threat, green monkeys produced alarm calls that were similar to the aerial alarm calls of closely related East African vervet monkeys (Wegdell et al. 2019). After a few such exposures, the sound of the drone was already sufficient for subjects to respond appropriately, i.e., by immediately scanning the sky and running for cover. Two further studies involving potentially dangerous terrestrial threats have also provided evidence for rapid, one-trial social learning in monkeys (Deshpande et al. 2022; León et al. 2022). There is also evidence for more gradual learning, mainly supported by older observational studies (Castro and Snowdon 2000: *Saguinus oedipus*; Fichtel 2008: *Propithecus verreauxi verreauxi*; Fischer et al. 2000: *Papio cynocephalus ursinus*; McCowan et al. 2001: *Saimiri sciureus*; Ramakrishnan and Coss 2000: *Macaca radiata*), although it is often unknown what sorts of experiences individuals have had throughout their early lives.

Whatever the mechanism, social learning is likely to be of key importance during acquisition. In a recent study, when infant vervet monkeys heard alarm calls, they were more likely to respond appropriately if they first looked at more experienced group members (Seyfarth and Cheney 1986; Mohr et al. 2022), a form of social referencing (Evans and Tomasello 1986; Baldwin and Moses 1996). Moreover, research on immature wild orangutans has shown that observational social learning by peering is a critical component of the acquisition of learned subsistence skills like feeding and nest-building (Schuppli et al. 2016).

Another important but often overlooked factor in ontogenetic studies of primate communication is that, under natural conditions, animals do not just interact with members of their own species, but usually operate in a network of associated species, with sometimes shared predators. This leads to opportunities for mutually beneficial antipredator efforts, especially if there are discrepancies in the abilities to detect predators (Seppänen et al. 2007; Goodale et al. 2010), but also for learning by eavesdropping on other species’ alarm calling. For example, arboreal species that forage in the upper canopy may be better at detecting aerial predators than species exploiting the lower vegetation (Morse 1977; Gautier-Hion et al. 1983; Munn 1986). Many species, and particularly forest primates, exploit this fact by forming mixed-species associations, a behavioural strategy that reduces predation risk (Whitesides 1989; Bshary and Noë 1997; Heymann and Buchanan-Smith 2000; Stensland et al. 2003; Oliveira and Dietz 2011). Alarm calling may play a key role in mediating the benefits of such mixed species associations. Primates respond well and appropriately to the alarm calls of other species (Ramakrishnan and Coss 2000; Wheeler and Hammerschmidt 2013; Di Bitetti and Wheeler 2017), regardless of taxonomic groups (Hauser 1988; Seyfarth and Cheney 1990; Zuberbühler 2000a; Seiler et al. 2013). For example, both Diana monkeys (*Cercopithecus diana*) and Campbell’s monkeys (*C. campbelli*) understand each other’s leopard and eagle alarm calls (Zuberbühler 2000b, 2001) and similar findings have been reported among prosimians (Oda and Masataka 1996; Oda 1998; Fichtel 2004) and platyrrhines (Wheeler et al. 2019).

Although predation is one of the main selection pressures, learning about predators during actual predation events can be dangerous, which poses the question of how animals can acquire alarm call competence if learning opportunities are costly. Moreover, though vocal production in non-human primates is predominantly innate, vocal usage and comprehension are influenced by learning (Seyfarth and Cheney 2010). This is even more pertinent for the comprehension of most heterospecific calls because it is unlikely that there is a genetic predisposition for understanding signals of unrelated species. However, there is little research comparing the development of con- and heterospecific alarm call comprehension in non-human primates, so much of these arguments are merely based on general plausibility. Notable exceptions are the studies conducted by Hauser (1988) and Fichtel (2008). In the first one, infant vervet monkeys did not recognize superb starling (*Lamprotornis superbus*) alarm calls at birth but there were individual differences in how fast juveniles learned to respond appropriately as a function of local starling densities (Hauser 1988). Similar findings have also been reported from young Verreaux’s sifakas learning red fronted lemur (*Eulemur fulvusrufus*) alarm calls. After only 4 months old, infant sifakas started to discriminate between alarm and non-alarm stimuli (parrot song) and adult-like responses to their own and red fronted lemur alarm calls appeared only after 6–8 months (Fichtel 2008). Overall, both studies presented evidence that alarm call competence depends on experience, but the details of how and when exactly young primates learn the meaning of other species’ alarm calls are unknown.

Here, we investigated the development of con- and heterospecific alarm call recognition in sooty mangabeys (*Cercocebus atys*; hereafter mangabeys), a terrestrial, forest-dwelling, West African monkey. In Taï Forest (Ivory Coast), mangabeys form temporary mixed-species associations with other primate species (around 60% of time), mostly with arboreal Diana monkeys (10–15% of time) (Höner et al. 1997; McGraw and Bshary 2002; McGraw et al. 2007), with whom they share the same predators (Shultz et al. 2004): leopards (*Panthera pardus*), crowned eagles (*Stephanoaetus coronatus*), chimpanzees (*Pan troglodytes*) (Boesch and Boesch-Achermann 2000; McGraw et al. 2007) and humans. Encounters with snakes are also common although snakes do not typically prey on mangabeys. Nevertheless, lethal accidental encounters occur, most often with highly venomous Gaboon (*Bitis gabonica*) and rhinoceros vipers (*B. nasicornis*) (Range and Fischer 2004). Overall estimated predation rates (proportion of population removed per year) by leopards, crowned eagles and chimpanzees are higher for terrestrial than for arboreal Taï forest prey (Shultz and Thomsett 2007). Moreover, leopard diets are biased towards terrestrial prey, whereas crowned eagle diets do not show a bias towards either terrestrial or arboreal prey (Shultz et al. 2004). Furthermore, the estimated crowned eagle, leopard and chimpanzee predation rates are around 13%, 6% and < 1% of the mangabey Taï population, respectively (Shultz et al. 2004).

The different predators differ in their hunting strategies and primates usually show adaptive responses to each predator. Reactions to leopards entail rapidly climbing into the lower forest canopy, followed by mobbing and leopard-specific alarm calls at high rates by some individuals, presumably to drive the predator away (Zuberbühler et al. 1999; Zuberbühler and Jenny 2002, 2007). For crowned eagles, mangabeys immediately seek cover or monitor the sky to keep visual contact, depending on the eagle’s behaviour, again while some individuals give eagle-specific alarm calls at high rates (Shultz and Thomsett 2007). For stationary snakes, such as Gaboon and rhinoceros vipers, mangabeys jump aside showing startle responses, followed by standing bipedally and cautiously approaching and scanning the area of the snake (Range and Fischer 2004; Penner et al. 2008), while some give snake-specific alarm calls that attract other individuals to the site. Finally, for chimpanzees, mangabeys adopt a cryptic behaviour and rapidly move away in silence (Zuberbühler et al. 1999). Diana monkeys not only produce acoustically distinct alarm calls in response to leopards and eagles, similar to mangabeys, but they also produce distinct calls for non-predatory disturbances (i.e., falling trees or branches) (Zuberbühler et al. 1997).

We are not aware of any systematic research on the development of con- and heterospecific alarm call comprehension in primates, apart from Hauser (1988) and Fichtel (2008). To address this gap, we combined natural observations with playback experiments to observe the response of mangabeys of three age classes—young juveniles (1–2 years), old juveniles (3–4 years), and adults (> 4 years)—to predator-specific alarm calls produced by their own group members or by sympatric Diana monkeys.

Similar to what has already been reported from vervet monkey alarm call development, we predicted that juveniles would show lower response rates to alarm calls and alarm call to a wider variety of stimuli than adults. We also predicted that social referencing, i.e., looking at other group members when hearing an alarm call, would play a key role in alarm call behaviour, especially in young individuals. Moreover, we predicted predator threat to be a major driver of development, with conspecific leopard alarms being established first in early development, because leopard predation has been linked to the evolution of primates’ cognitive flexibility (Zuberbühler and Jenny 2002). Regarding heterospecific alarms, i.e., the alarm calls of Diana monkeys, we predicted that mangabeys would first discriminate predatory (leopard and eagle alarms) from non-predatory threats (falling tree alarms), as seen in other species (Cheney and Seyfarth 1990; Fichtel 2008), before learning to discriminate specifically between Diana monkeys’ eagle and leopard alarms. Finally, because primates are unlikely predisposed to respond to alarm calls of unrelated species and because mangabeys probably witness fewer predation events on Diana monkeys than on their own group members —having less opportunities to make specific predator-alarm calls associations, we predicted faster learning of conspecific than heterospecific alarm calls.

## Methods

### Study site and species

The study was conducted from May to December 2018 and August 2019 to March 2020 with two groups of free-ranging mangabeys in Taï National Park, Ivory Coast. During the study period, we conducted 84 trials on the main group (TCP mangabeys) whose size ranged between 74–91 individuals, including 23 adult females (> 5 years old), 7 adult males (> 7 years old), 6 subadult females (4–5 years old), 6 subadult males (5–7 years old), 9 old juvenile females (3–4 years old), 8 old juvenile males (3–5 years old), 10 young juvenile females (1–2 years old), 12 young juvenile males (1–2 years old) and 10 infants (< 1 years old) (McGraw and Zuberbühler 2007). We also conducted 28 trials on a non-neighbouring group (TMP mangabeys) whose home range was about 4 km Northwest and which included 62–67 individuals, including 24–26 adults, 9 subadults and 20 juveniles and 9–12 infants (Wittig 2018; Mielke et al. 2019). Both groups were fully habituated to human observers and have been under study for several years.

### Playback stimuli

Playback stimuli were obtained at the study site by recording alarm calls occurring during encounters with real predators, leopard and snake models and playbacks of leopard growls and eagle shrieks. Recordings were made with a Marantz PMD 661 MKII digital recorder and an MKH 416-P48U3 Sennheiser directional microphone. Sound files were stored and processed as.wav files with 44.1 kHz sampling rate, 16 bits amplitude resolution using Audacity 2.2.2 (Audacity Team 2020) and Raven 1.4 software (Center for Conservation Bioacoustics 2014). Recordings were screened for exemplars with low signal-to-noise ratio, absence of signal overlap and recorded at distances from 4–15 m. Playback stimuli were edited such that each consisted of three alarm call sequences with intervals of 5 s silence in between, trying to mimic natural alarm call sequences. We used 43 alarm calls produced by 25 mangabeys for leopards (*N* = 15), crowned eagles (*N* = 14) and vipers (*N* = 14) as conspecific stimuli (Fig. S1), and 57 alarm calls produced by 21 male Diana monkeys for leopards (*N* = 16), eagles (*N* = 22) and falling trees (*N* = 21) as heterospecific stimuli (Fig. S2). No stimulus was used in more than two trials. We did not include responses to chimpanzees because monkeys adopt a cryptic behaviour in response to them and chimpanzees rarely prey on mangabeys.

### Playback procedures

We conducted a total of 112 playback trials (*N* = 49 conspecific; *N* = 63 heterospecific) on 15 young juveniles, 16 old juveniles and 18 adults (see Table S1 and Supplementary data). Thirty six (*N* = 36) subjects were used in more than one trial (2–4 trials), yet subjects were never tested more than once for each condition. To avoid pseudo-replication, we used vocalizations from different adult individuals as playback stimuli where possible, and never used the same stimulus nor the same call provider twice on a subject. Moreover, for the 10 stimuli that were used twice, we ensured that the same stimulus was not played more than once during the same month to prevent habituation effects. Finally, we excluded nine conspecific trials from analysis (*N* = 2 young and *N* = 5 old juveniles, and *N* = 2 adults) because other monkey species started alarm calling before the subject reacted and three more heterospecific trials were aborted because of a technical failure (*N* = 2 young juveniles and *N* = 1 adult).

Mangabeys commonly hear alarm calls of other group members and Diana monkeys in the study area (Table S2), suggesting that two trials per week, one from each species, was well within the monkeys’ natural range of experience. For each conspecific trial, we took care to always broadcast a call from a call provider that was in the same social group, but out of sight at the moment of the experiment. Subjects were tested in a randomized but counterbalanced order, and when they were alone or in small parties and engaged in quiet activities (e.g., foraging, resting or auto-grooming).

Two experimenters were needed to carry out a playback trial. Experimenter 1 followed and filmed the focal subject using a Panasonic HC-V500 camera continuously, before (~ 30 s), during and after the playback (~ 30 s or as long as possible). Experimenter 2 predicted the subject path and strategically positioned the playback equipment around 5–10 m away from the subject, hidden behind buttress roots out of sight of individuals. To emulate natural conditions, the speaker was positioned on the ground or on elevated locations, such as trunks of fallen trees or small hills, during con- and heterospecific trials, respectively. Before each trial, the focal subject was followed for 15 min prior to starting the playback to ensure there were no external stimuli modifying his/her behaviour. We proceeded to broadcast the playback stimulus if no alarm calls were produced during the hour before, neither by the any member of the studied group or any other monkey species. Subjects were never more than 2 m high during the experiments.

All stimuli were broadcasted using an Apple iPod touch digital player connected to an AER alpha speaker amplifier. We used a Dostmann MS-85 (Dostmann) mini-amplitude level meter to adjust the sound level. Absolute amplitude levels of the different stimuli variated between 95–103 dB(C) and 81–85 dB(C) for mangabey calls produce in response to leopards and eagles, and snakes, respectively, and 99–107 dB(C) for Diana monkey calls, measured at 50 cm from the speaker, to match natural characteristics of the different calls.

### Independent variables

For every trial, we noted subject and caller provider identity, caller provider species, alarm call type, using stratum and audience composition within a 10 m radius of the subject. We considered a subject to be alone when no other group members were present within that distance. We used BORIS coder (Friard and Gamba 2016; www.boris.unito.it) to analyse video recordings on a frame-by-frame basis (25 frames s^–1^) during the first 30 s after model detection.

### Behavioural response variables

We first scored the occurrence of predator-specific behavioural responses to leopards, eagles and snakes (Table 1). Then, we classified a behavioural response as “appropriate” if it matched the corresponding predator-specific alarm call used as stimulus. For Diana monkey loud calls given to trees the absence of antipredator behaviours was considered as the appropriate response. Additionally, we registered the number and type of calls emitted by the focal individual when vocal responses occurred.

**Table 1:** Definitions and predictions of behavioural responses

Behavioural response	Definition
Leopard antipredator	Subject climbs into a tree, flees and/or emits leopard alarm calls
Eagle antipredator	Subject looks for cover or runs down trees, constantly scans the sky and/or emits eagle alarm calls
Snake antipredator	Subject stays and scans the forest floor, approaches and inspects, jumps aside, stands bipedally and/or emits snake alarm calls
No antipredator response	Subject shows no particular antipredator response and continues engage in his/her activity previous the experiment

To explore whether juveniles copied or looked for clues among other individuals when not knowing how to respond to the stimulus (see Seyfarth and Cheney 1986; Fichtel 2008), we also counted the number of subjects that looked at adults as response to the playback experiments as a form of social referencing.

It was not possible to record data blind because our study involved trials on focal animals in the field. However, to minimize observer bias, blinded methods were used when behavioral data were analyzed: to estimate observer reliability, JL and CT separately blind-coded (65/100) 65% of the trials, resulting in a very good inter-rater reliability (Cohen's kappa for appropriate antipredator behaviour K = 0.91 and for social referencing: K = 1).

### Natural stimuli eliciting alarm calls

We followed the main study group from dawn to dusk and used 20-min focal animal samples (Altmann 1974) to record detailed behavioural data for all members of the group. We registered 930 focal samples (number of focal samples: adults N = 531; subadults N = 126; old juveniles N = 138, young juveniles N = 135). During each focal sample, we recorded data on the stimuli that elicited different types of alarm calls by the focal subject using an all-occurrence sampling. These stimuli were either the antipredator behaviour, usually alarm calls, exhibited by another group member or an heterospecific close by, the sight of a predator or the occurrence of a potential threat. We classified the species (or objects) that elicited alarm calls as confirmed predators (cp): defined as animals that prey on mangabeys; potential predators (pp): defined as animals that prey on species the size of mangabeys but are seldom observed to attack monkeys; confirmed threat (ct): defined as animals that are a lethal threat to mangabeys; potential threat (pt): defined as animals that can potentially pose a threat to mangabeys; non-threating (nt): defined as animals or objects that elicited alarm calls but do not pose a likely threat to mangabeys. Since an individual’s alarm calling is affected by the alarm calls of others, we only analysed data on the first alarm given in any alarming bout. We considered bouts of alarming to be independent after intervals of 15 min with no alarm calls. If more than one individual alarmed simultaneously at the start of a bout, all such callers were scored as first callers. The type of alarm call was identified by ear. We chose the first focal subject opportunistically and then sampled all individuals of the same age-sex class before making repeated samples of the same individual. No subsequent samples on the same individual were made less than 1 h apart from the previous focal sample.

### Statistical analysis

To investigate which factors had an impact on mangabey antipredator behavioural responses, we used a series of Binomial Generalized Linear Mixed Models (Bolker et al. 2009) using R version 4.0.3 (R Core Team 2020) and the function ‘glmer’ of the package lme4 (Bates et al. 2015). We tested appropriate behavioural response as the response variable in three separate models. The first two models had a con- and a heterospecific approach, respectively, by analysing the response variable against the same set of four fixed effects predictor variables: Playback stimulus (*conspecific model:* leopard, eagle or snake; *heterospecific model:* leopard, eagle, or falling tree), Age (young and old juvenile or adult), Stratum (ground or understory), and Audience (alone or in company). The third model had an interspecific approach and analysed the response variable against a dataset considering only alarm calls shared by both caller species (leopard and eagle alarm calls). For this last model, ‘Caller species’ (sooty mangabey or Diana monkey) was included as an additional fixed factor. ‘Playback stimulus’, ‘Age’ and ‘Caller species’ were our main variables of interest and were considered the test predictors, with ‘Stratum’ and ‘Audience’ considered to be control predictors. Moreover, ‘Subject’ and ‘Caller identity’ were taken as random factors in all models to account for repeated measurements. To check whether the control predictors drove the results (Simmons et al. 2011), we re-ran all the analyses without it. The results were robust. Spatial autocorrelation was tested for each model and when an effect was detected it was corrected in the model. Finally, for all models, we checked for over-dispersion.

To test the significance of the fixed factors and their relations, we used the ‘Anova’ function (car package) in each model to perform a type III or II ANOVA Wald Chi-Square Tests, depending on whether or not there was a significant interaction in the model. Originally, all explanatory variables and interactions involving the test predictors were integrated into the full models. Then, insignificant interactions were removed to simplify the model (Engqvist 2005). The significance threshold α related to the test predictors was set at 0.05. We then conducted pairwise post hoc comparisons between levels of statistically significant control predictors by computing estimated marginal means for each model, using the ‘emmeans’ function and package. For these comparisons, we included a Tukey honest significant difference adjustment to account for running multiple tests on the same data. We also conducted binomial tests to analyse the vocal response of the subjects during the trials. Because of the low number of alarm calls given as response (N = 7), we could not run any further analyses. Finally, we analysed whether juveniles and subadults were less selective in their alarm calling as compared with adults by comparing the observed distributions of immatures alarm calls with the distributions that would have been expected if immatures had produced their alarms exactly like adults did during natural alarm calling events by goodness-of-fit tests. When expected counts were too small, we estimated *P* values using Monte Carlo simulations based on 10,000 permutations of the original data set to properly perform goodness-of-fit tests (option “simulate.p.value = TRUE” in the R chisq.test function) (Verzani 2004).

## Results

### Call usage: vocal responses during natural encounters

During 310 h of focal animal data, we registered 91 alarm calls in 86 natural predator encounters in which focal subjects were the first individuals to call. In five of those encounters two individuals gave the first alarm calls simultaneously. Encounters with leopards, crowned eagles, dangerous snakes (pythons and vipers: N = 45) and potential dangers (civets, genets, dwarf crocodiles, large-sized non-carnivorous mammals; N = 23) accounted for 73.3% of the events. The remaining alarm calls were given during 23 encounters with animals and objects that probably were not a threat to them.

We found that alarm calls given for non-threatening stimuli and potential threats decreased with age. After correcting for the number of hours of focal animal data in each age class, young juveniles were the most likely individuals to give first alarm calls (number of first alarm calls per hour: young juveniles: 0.58; old juveniles: 0.37; subadults: 0.45; adults: 0.16). However, young juveniles produced more than half of their alarm calls in response to animals and objects that were unlikely to pose a threat to them (Table 2). In contrast, most of the alarm calls produced by subadults and adults were given in response to confirmed predators and threats to mangabeys.

**Table 2:** Distribution of alarm calls produced by immatures, compared with the distribution of alarm calls that would have been expected if immatures had distributed their alarm calls as adults did. Expected values are in parentheses

	*N* of alarm calls in response to			
	Confirmed dangers	Potential dangers	Unlikely threats	Significance
Young juveniles (*N* = 26)	3 *(20.6)*	7 *(4.5)*	16 *(0.9)*	*χ*^2^ = 268.8, *P* < 0.001
Old juveniles (*N* = 17)	6 *(13.5)*	6 *(2.9)*	5 *(0.6)*	*χ*^2^ = 40.33, *P* < 0.001
Subadults (*N* = 19)	13 *(15.1)*	5 *(3.3)*	1 *(0.7)*	*χ*^2^ = 1.37, *P* = 0.54
Adults (*N* = 29)	23	5	1	–

All individuals produced each type of alarm call in non-arbitrary ways. Leopard alarm calls were restricted in response to carnivores and terrestrial mammals, usually of medium to large body size (Table 3). Moreover, eagle alarm calls were only given for avian species, while snakes alarm calls were elicited by reptiles, mostly snakes, and animals and objects that resemble the colour or the shape of vipers, i.e., toads and small logs.

**Table 3:** Number of times individuals in each age class gave an alarm call for different species and objects during focal follows. Number of hours of focal animal data for each age class are in parentheses. Species are listed by body size in each category (data from: mammals: Kingdon (2015); birds: Borrow (2014); reptiles: Chippaux (2006) and Trape et al. (2012)). Cp: confirmed predator, Pp: potential predator, Ct: confirmed threat, Pt: potential threat, Nt: non-threating

			*N calling events*			
Scientific name	Common name	Threat	Adults (177 h)	Subadults (42 h)	Old juveniles (46 h)	Young juveniles (45 h)
Leopard alarm call for carnivores						
*Panthera pardus*	Leopard	Cp	2	1	1	
*Civettictis civetta*	African civet	Pp	1		1	
*Genetta pardina*	Genet	Pt	1		2	2
Leopard alarm call for other mammals						
*Hylochoerus meinertzhageni*	Giant forest hog	Pt		1	1	
*Hexaprotodon liberiensis*	Pigmy hippopotamus	Pt	1	1		2
*Cephalophus jentinki*	Jentink’s duiker	Pt		1	1	1
*Potamochoerus porcus*	Red river hog	Pt	1	1		1
*Cephalophus dorsalis*	Bay duiker	Nt			1	2
*Cephalophus niger*	Blacked duiker	Nt				1
*Cephalophus* (undet.)	Unknown duikers	Nt			1	2
Eagle alarm call for birds						
*Stephanoaetus coronatus*	African crowned eagle	Cp	15	7	3	1
*Ceratogymna atrata*	Black-casqued hornbill	Nt	1		1	3
Strigidae (undet.)	Unknown small-size owl	Pt				1
Snake alarm call for reptiles, amphibians and vegetation						
*Python regius*	Python	Cp			1	
*Osteolaemus tetraspis*	African dwarf crocodile	Pt	1	1	1	
*Bitis gabonica / B. rhinoceros*	Gaboon viper / Rhinoceros viper	Ct	6	5	1	2
Colubridae (undet.)	Unknown small-size colubrids	Nt			1	4
*Sclerophrys* (undet.)	Unknown toad	Nt				1
–	Small logs and dry leaves on the ground	Nt		1	1	3

However, the number of species classified within these broad categories varied considerably among age classes (Table 3). Juveniles gave leopard alarm calls for at least ten different species, whereas adults produced leopard alarm calls to five. In a similar way, juveniles gave snake alarm calls in response to seven different species or objects, whereas adults restricted snake alarm calls to Gaboon and rhinoceros vipers. Overall, we found that juveniles but not subadults were less selective than adults in their alarm call behaviour. When giving alarm calls, juveniles were significantly less likely to restrict alarms to confirmed predators and threats, and significantly more likely to give alarm calls for potential dangers and, in particular, for non-threatening animals (Table 2).

### Call usage: vocal responses during experiments

Six (N = 6) responded with their own alarm calls to alarm call playbacks in seven (N = 7) of 100 trials (7%) (leopard alarms: conspecific 3 of 13; heterospecific 2 of 17; eagle alarms: conspecific: 1 of 12; heterospecific 0 of 21; snake alarms: conspecific: 1 of 15; tree alarms: heterospecific 0 of 22; Supplementary data), always with the correct semantic category of the alarm call they had just heard (Binomial test (0.5): *P* = 0.007). All subjects were adults (N = 4 females, N = 2 males; Binomial test (0.5): *P* = 0.015) and all were on the ground (Binomial test (0.5): *P* = 0.007). Neither caller species nor audience drove the vocal response of the callers (Binomial test (0.5): Caller Species *P* = 0.226; Audience *P* = 0.226). Finally, the call providers during these trials were always different and did not follow any evident categorization pattern (e.g., sex, ranking pattern).

### Call comprehension: non-vocal responses during experiments (conspecific alarms)

**Fig. 1:**
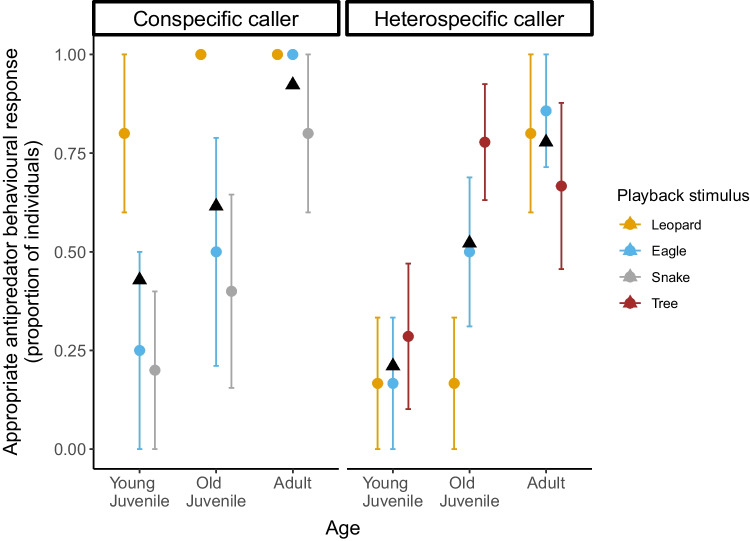
Development of con- and heterospecific alarm call behaviour: Proportion of individuals that showed appropriate specific antipredator behavioural responses to con- and heterospecific alarm call playbacks by age. Colour dots indicate mean values ± SE for leopard (yellow), eagle (blue), snake (grey), and tree (brown) playback stimuli. Black triangles indicate mean values for each age group’ corresponding response

In 40 of 49 trials, we were able to code the subjects’ antipredator responses to conspecific alarm call playbacks. In the ‘conspecific model’, we found that the test predictors ‘Age’ (*χ*^2^_2_ = 7.47, *P* = 0.023) and ‘Playback Stimulus’ (*χ*^2^_2_ = 6.679, *P* = 0.035) had an influence on the display of appropriate antipredator behavioural responses (Table 4a), with adults showing more corresponding specific antipredator behaviours than young juveniles (*z* = 2.733, *P* = 0.017; proportion of subjects mean ± SE: adults 0.92 ± 0.07 vs young juveniles 0.42 ± 0.13; Fig. 1; Supplementary video-clips S1, S2). Likewise, conspecific leopard alarm calls elicited more corresponding specific antipredator behaviours than conspecific snakes alarm calls (*z* = 2.568, *P* = 0.027; proportion of subjects mean ± SE: leopard alarm 0.92 ± 0.07 vs snake alarm 0.46 ± 0.13; Fig. S3).

**Table 4:** Influence of predictor variables on behavioural responses

Predictor variable	Estimates	SE	P*
a. Conspecific model			
Age			**0.023**
*Age (old juvenile)*	* − 2.19*	*1.29*	
*Age (young juvenile)*	* − 4.12*	*1.5*	
Playback stimulus			**0.035**
*Playback stimulus (eagle)*	* − 3.13*	*1.46*	
*Playback stimulus (snake)*	* − 3.87*	*1.5*	
Stratum			0.876
*Stratum (understory)*	*0.2*	*1.33*	
Audience			0.276
*Audience (yes)*	*1.19*	*1.09*	
b. Heterospecific model			
Age			**0.004**
*Age (old juvenile)*	* − 1.59*	*0.78*	
*Age (young juvenile)*	* − 2.87*	*0.88*	
Playback stimulus			0.208
*Playback stimulus (eagle)*	*0.66*	*0.95*	
*Playback stimulus (tree)*	*1.43*	*0.81*	
Stratum			0.157
*Stratum (understory)*	*1.13*	*0.8*	
Audience			0.808
*Audience (yes)*	* − 0.16*	*0.68*	
c. Interspecific model			
Caller species			**0.001**
*Caller* s*pecies (Diana monkey)*	* − 5.02*	*1.56*	
Age			**0.001**
*Age (old juvenile)*	* − 2.98*	*1.05*	
*Age (young juvenile)*	* − 3.92*	*1.1*	
Playback stimulus			**0.02**
*Playback stimulus (eagle)*	* − 3.27*	*1.41*	
Stratum			0.065
*Stratum (understory)*	*1.89*	*1.03*	
Audience			0.082
*Audience (yes)*	*1.56*	*0.89*	
Caller species * Playback stimulus			**0.011**
*Caller* species (Diana monkey)*Playback stimulus (eagle)	*4.48*	*1.77*	

() denote the variable level that reflects the estimate when tested against the alternative level: Eagle and Snake vs Leopard, Old and Young juvenile vs Adult, Understory vs Ground, Audience vs Alone, Diana monkey vs Sooty Mangabey

^*^The critical *P* value related to the test predictors was set at 0.05

### Call comprehension: non-vocal responses during experiments (heterospecific alarms)

In 60 of 63 trials, we were able to code subjects’ antipredator behavioural response to Diana monkey alarm call playbacks. In the ‘heterospecific model’, we found that the test predictor ‘Age’ (*χ*^2^_2_ = 10.68, *P* = 0.004) had an influence on the display of appropriate antipredator behavioural responses (Table 4b). Pairwise comparisons revealed that adults showed more corresponding specific antipredator behaviours as response to heterospecific alarm calls than young juveniles (*z* = 3.268, *P* = 0.003; proportion of subjects mean ± SE: adults 0.77 ± 0.1 vs young juveniles 0.21 ± 0.09; Fig. 1; Supplementary video-clip S3).

### Call comprehension: interspecies comparisons

**Fig. 2:**
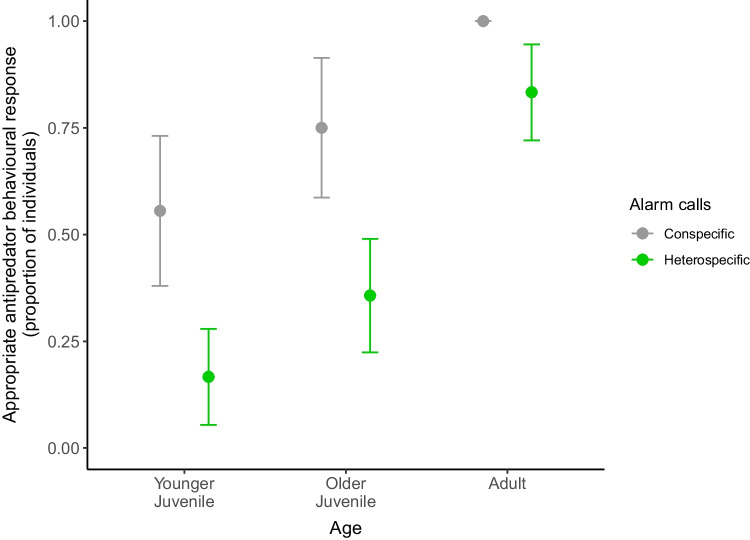
Development of con- and heterospecific alarm call behaviour: Proportion of individuals that showed appropriate specific antipredator behavioural responses to leopard and eagle alarm call playbacks from con- and heterospecifics by age. Colour dots indicate mean values ± SE for conspecific (grey), and heterospecific (green) playback stimuli

**Fig. 3:**
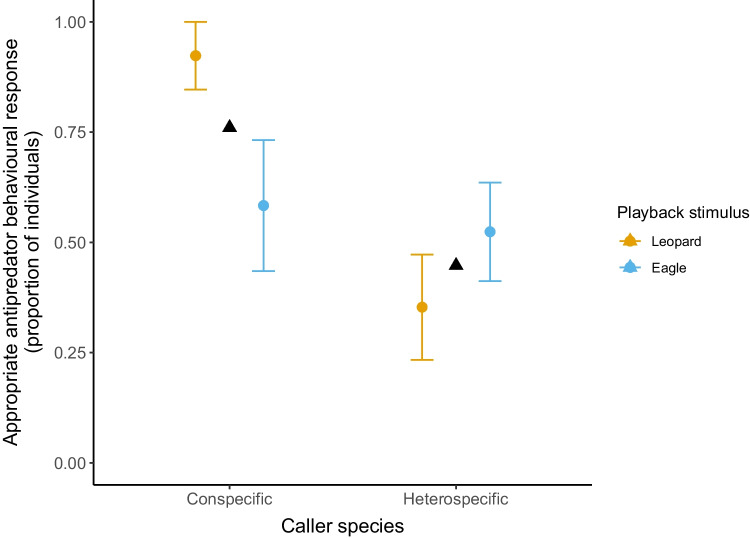
Interspecies comparison: Proportion of individuals that showed appropriate specific antipredator behavioural responses to leopard and eagle alarm call playbacks depending on the caller species. Colour dots indicate mean values ± SE for leopard (orange) and eagle (blue) playback stimuli. Black triangles indicate corresponding response mean values to con- and heterospecific alarm calls

In the interspecific model, we evaluated subjects’ antipredator behavioural responses to mangabeys’ and Diana monkeys’ leopard and eagle alarm call playbacks (N = 63 trials) and found that the test predictor ‘Age’ (*χ*^2^_2_ = 12.86, *P* = 0.001) had an influence on the display of appropriate antipredator behavioural responses (Table 4c). Adults showed more corresponding specific antipredator behaviours as response to alarm calls of both species than juveniles (Old juveniles:* z* = 2.814, *P* = 0.013; proportion of subjects mean ± SE: adults 0.9 ± 0.06 vs old juveniles 0.5 ± 0.11; Young juveniles: *z* = 3.544, *P* = 0.001; proportion of subjects mean ± SE: adults 0.9 ± 0.06 vs young juveniles 0.33 ± 0.1; Figs. 1, 2). Moreover, we found a significant interaction between ‘Playback stimulus’ and ‘Caller Species’ (*χ*^2^_1_ = 6.381, *P* = 0.011) (Table 4c). Post hoc tests showed that leopard alarm calls produced by conspecifics elicited more corresponding specific antipredator behaviours than the ones given by heterospecifics in response to both leopards and eagles (Heterospecific leopard alarms:* z* = 3.205, *P* = 0.007; proportion of subjects mean ± SE: conspecific leopard alarms 0.92 ± 0.07 vs heterospecific leopard alarms 0.35 ± 0.12; Heterospecific eagle alarms: *z* = 2.731, *P* = 0.032; proportion of subjects mean ± SE: conspecific leopard alarms 0.92 ± 0.07 vs heterospecific eagle alarms 0.52 ± 0.11; Fig. 3).

### Social referencing

We found that for both con- and heterospecific alarm calls the number of individuals looking toward an adult just after hearing an alarm call decreased with age (Supplementary video-clip S4). When hearing conspecific alarm calls, young and old juveniles looked towards adults in 6 of 14 (42.9%) and 4 of 13 trials (30.8%), respectively, whereas no adult showed this response. Similarly, during heterospecific alarm call trials, young and old juveniles looked towards adults in 10 of 19 (52.6%) and 5 of 23 trials (21.7%), respectively, whereas adults did it in 3 of 18 trials (16.7%; Table 5). Because of low sample size, we could not run any further statistical analyses.

**Table 5:** Number of individuals that showed antipredator behavioural responses with respect to the stimulus heard

	Antipredator response		
	correct	look at adult	incorrect
*Conspecific alarm calls*			
Young juveniles (*N* = 14)	6 (42.9%)	6 (42.9%)	2 (14.3%)
Old juveniles (*N* = 13)	8 (61.5%)	4 (30.8%)	1 (7.7%)
Adults (*N* = 13)	12 (92.3%)	0 (0.0%)	1 (7.7%)
*Heterospecific alarm calls*			
Young juveniles (*N* = 19)	4 (21.1%)	10 (52.6%)	5 (26.3%)
Old juveniles (*N* = 23)	12 (52.2%)	5 (21.7%)	6 (26.1%)
Adults (*N* = 18)	14 (77.8%)	3 (16.7%)	1 (5.5%)

## Discussion

Sooty mangabeys show specific behavioural responses, including predator-specific alarm calls, to their predators, but little is known about the development of such behaviour. In this study, we examined the development of alarm call behaviour during natural encounters and in response to experimentally presented conspecific and heterospecific alarm calls. Generally, we found increasing competence with age, both in terms of alarm call comprehension, usage and production. Across conditions, young juveniles (12 to 24 months old) showed the poorest performance and were most likely to engage in social referencing. Old juveniles (24 to 48 months), on the other hand, already showed responses that did not differ significantly from adults, suggesting that the main learning events mainly happen in the first 24 months of their lives. Adults differed from the juveniles insofar as they were particularly sensitive to others’ alarm calls and were more likely to respond with corresponding antipredator behaviours, including by responding with their own alarm calls. Mangabeys classified species and objects from the world around them into broad categories since a young age (Table 3). However, adults were more selective than juveniles in their alarm calling (Table 2). In sum, these results suggest that in mangabeys alarm call comprehension becomes entrenched during the first 2 years of life, while competent alarm call usage does not appear until about 4 years of age, suggesting that comprehension precedes usage and production in the alarm call system of mangabeys.

Across predator types, leopard alarm calls elicited the strongest overall responses in all age classes, while snake alarm calls only prompted mild responses in juveniles in both con- and heterospecific conditions. Alarm call vocal responses, specifically, depended on whether the caller was a con- or heterospecific and the predator reference. Mangabey leopard alarms elicited more corresponding antipredator behaviours than both Diana monkeys’ leopard and eagle alarm calls, highlighting the importance of leopard predation pressure in shaping mangabeys’ alarm call behaviour. Finally, we also found no clear differences in learning between predatory and non-predatory Diana monkey loud calls nor of con- and heterospecific alarm call signal meaning.

Our findings align with previous work that has shown that — despite some degree of predisposition towards discriminating between predator-specific alarm calls — experience and social input are essential for the development of primate alarm call behaviour (Fichtel 2008: Sifakas, *Propithecus verreauxi verreauxi*; Fischer et al. 2000: baboons, *Papio cynocephalus ursinus*; McCowan et al. 2001: squirrel monkeys, *Saimiri sciureus*; Hauser 1988; Seyfarth and Cheney 1980, 1986: vervet monkeys, *Cercopithecus aethiops*). Learning appears to begin as soon as infants start moving independently at around 6 months. However, our results suggest that the full development of adult-like responses to alarm calls in mangabeys requires at least 2 years, which is longer than what has been reported for other primates (Seyfarth and Cheney 1980, 1986; Fischer et al. 2000; McCowan et al. 2001; Fichtel 2008). The fact that we took into account not only social referencing behaviour that emerges early, but also conspicuous locomotor and vocal responses could account for some, but not all, of this discrepancy. Moreover, similar to findings in vervet monkeys ( Seyfarth and Cheney 1986; Mohr et al. 2022), it is likely that social referencing plays an important role in how unexperienced individuals learn to respond to alarm calls. Across conditions, looking towards an adult when hearing an alarm call was the most common response in young juveniles (Table 5), suggesting that they were gathering information from more experienced individuals about how to respond to alarm calls. A similar behaviour, social peering, has been validated as an index of observational and social learning in young wild orangutans (Schuppli et al. 2016).

When analysing call usage during natural encounters with predators, we found that juveniles were more reactive and less selective in their calling behaviour, producing alarm calls far more often and to a wider variety of species and objects, than adults. However, this lack of selectivity of stimuli-signal associations was not arbitrary, analogous to young children’s overextension of early use of words (Rescorla 1980; Clark 2003). Juveniles produced leopard alarms almost exclusively to carnivores and medium to large body size terrestrial mammals, eagle alarms for birds and snake alarms for reptiles and snake-like animals and objects. Moreover, this broad categorisation appeared to be based not only on stimuli appearance but also on its behaviour and, possibly, its potential to pose a danger to the monkeys. For example, mangabeys, including adults, use leopard alarms as a warning mechanism in response to animals that, due to their body size, could pose a threat to a mangabey when charging or running around aimlessly (e.g., giant forest hogs, pigmy hippopotamus, large-sized duikers, herd of red river hogs) (Table 3). Another example of possible categorisation by overextension is the observation of a juvenile giving an eagle alarm after seeing a Dwarf galago (*Galagoides demidovii*) flying nearby (Clémentine Bodin personal communication). These differences in the usage of alarm calls between juveniles and adults may be related to the higher predation risks and lack of experience in dealing with predators of the former (Wrangham and Cheney 1985; Janson and van Schaik 1993; Isbell 1994).

Overall, these results provide evidence that over the course of their first four years of life, mangabeys reduce and refine their alarm calling behaviour to relevant predator species and dangerous contexts. Furthermore, our findings align with the gradual development of alarm call usage in vervet monkeys (Seyfarth and Cheney 1986; Cheney and Seyfarth 1990). Thus, while non-primate vocalizations are in many ways fundamentally different from human language and speech, future research should address how limited are the analogies between the ontogeny of alarm call usage in monkeys and the acquisition of communicative competence in young children.

When analysing call usage during playback experiments, we found age and location had an important effect on alarm calling behaviour as only adults that were foraging on the ground alarm called to the playbacks. Although further research is needed, this suggests that costly alarm calling requires general maturational processes, full integration into the group’s social and kin networks and parental status (e.g., access to mating partners, survival of socially important individuals and its kin, and enhance likelihood to sire offspring) (Cheney and Seyfarth 1990; Haff and Magrath 2013; Stephan and Zuberbühler 2021; Quintero et al. 2022). Alternatively, and not necessarily mutually exclusive, the small number of vocal responses to the playbacks could indicate that it is the predator-call association, and not alarm calls alone, that elicits strong responses, including alarm call behaviour (Owren and Rendall 2001; Ducheminsky et al. 2014). The finding that callers were on the ground at the moment of calling was simply a consequence of the mangabeys’ terrestrial foraging habits and the fact they could only encounter vipers on the floor of the forest.

As expected, conspecific leopard alarm calls elicited the strongest responses across age classes. Additionally, mangabeys’ leopard alarm calls elicited more corresponding antipredator behaviours than both Diana monkeys’ leopard and eagle alarm calls (Fig. 3). In the Taï forest, leopards often prey on mangabeys, probably because these are medium-sized terrestrial monkeys living in large groups, which are easier for the leopards to locate and ambush successfully than other prey (Zuberbühler and Jenny 2002). During focal follows, we observed that young mangabeys react to most events or signals (e.g., alarm calls) that generate arousal with a default reaction, which includes fleeing by jumping and climbing into the nearest tree, a behaviour that is effective for escaping a leopard attack. Moreover, mangabeys are better sentinels for ground predators than any other monkey species in the Taï forest and can spot a leopard visual model at a distance of up to 40 m (McGraw and Bshary 2002). Furthermore, field experiments have shown that experience with leopards is not required for Guereza colobus monkeys to produce antipredator-specific responses (Schel and Zuberbühler 2009). Our results suggest that young mangabeys seem to exhibit a predetermined reaction to threats as a hardwired evolutionary adaptation to produce better survival rates during leopard attacks. Overall, the observed patterns of behaviour in response to leopard-related stimuli supports the idea that leopard predation seems to have had a significant effect on primates’ cognitive evolution (Zuberbühler and Jenny 2002).

On the other hand, contrary to conspecific leopard alarm calls, snake alarm calls only prompted strong responses from adults (Fig. 1). While the predetermined behaviour of juveniles described above is adaptative for a leopard attack, it is likely an overreaction for a viper snake encounter, wherein the predator relies on short distance infrared imaging to detect prey and is not fast-moving over distance (Foerster 2008; Penner et al. 2008; Goris 2011). Mangabeys’ snake-specific antipredator behaviour appears to be complex: Because of their behavioural ecology and morphology, snakes may be more difficult to detect than other predators (Etting et al. 2014). Additionally, although 50–60 snake species can be found in the Taï forest region (Rödel and Mahsberg 2000; Ernst and Rödel 2002), only pythons and Gaboon and rhinoceros vipers elicit antipredator-specific responses from mangabeys. Hence, showing proper snake antipredator behaviours requires not only the recognition of snakes as predatory disturbances but also differentiation between the different snake types to distinguish the dangerous ones. Overall, it appears that the mild responses of juveniles for snake alarm calls are a consequence of mangabeys perceiving vipers as a less threatening and more complex danger than leopards, suggesting that snake antipredator behaviour requires more experience to be fully acquired than responses to other predators.

Our results showed that mangabeys are sensitive to the predator-specific alarm calls of Diana monkeys and respond to them as if the corresponding predator was present (Fig. 1). However, contrary to our predictions, there was no clear difference in the learning of predatory and non-predatory Diana monkey loud calls. A possible explanation could be found in the acoustic structure of these calls. Although loud calls given to falling trees tend to elicit calls with more syllables compared to leopard loud alarm calls (median number of syllables per call: 7 (range 1–16) vs. 3 (range 1–33), respectively), their general acoustic structure is very similar, which could create certain ambiguity between them (Zuberbühler et al. 1997). Thus, it might be possible that Diana monkeys’ tree loud calls could be difficult to distinguish from the loud alarm calls given for leopards and therefore, young individuals may require enough experience to learn how to distinguish them accurately.

Although mangabeys of all age-groups were more likely to respond to conspecific alarm calls than to Diana monkey calls (Fig. 1), this effect seemed to be driven by the strong responses given to conspecific leopard alarm calls. Indeed, we found no difference in con- and heterospecific signal meaning learning (Fig. 2). Functional semanticity of alarm calls of both species was acquired during juvenile stage, with adults showing higher response rates to con- and heterospecific alarm calls than both juveniles age classes, who presented considerable variation in their responses. This finding supports the notion that there should exist little genetic predisposition to comprehend heterospecific's alarm calls. Therefore, a similar pattern in the development of comprehension between con- and heterospecific alarm calls might be seen as a sign that that the underlying mechanism in the ontogeny of vocal comprenhension is learning rather than simple maturation. Similar development rates of responses to con- and heterospecific alarm calls has also been shown in Verreaux’s sifakas —albeit at a much earlier age (6–7 months old) (Fichtel 2008). Thus, it might be possible that the full appearance of adult-like responses to con- and heterospecific alarm calls in primates is mediated by similar learning process mechanisms, which could have species-specific learning parameters.

Of further interest is the extent to which primates are predisposed from birth to respond to their alarm calls, and how they learn the meaning of their alarm calls. In most primate species alarm calls are short with abrupt onsets and broadband noisy spectra (Rendall et al. 2009). Furthermore, studies on the vocalizations of African green monkeys (*Chlorocebus*) revealed that male barks of closely related species and subspecies are highly conserved in their acoustic structure (Price et al. 2014, 2015). However, mangabeys and Diana monkeys share a most recent common ancestor some 14.1 million years ago and are grouped in different tribes of the subfamily Cercopithecinae (Pozzi et al. 2014). Therefore, it is likely that mangabeys’ comprehension of indirect signs, such as Diana monkey alarm calls, requires considerable learning rather than being largely predisposed from birth. On the other hand, morphological computation research in infant common marmosets has demonstrated how changes in body morphology (lung growth) refine vocal usage over time (Zhang and Ghazanfar 2018). Our results show that the development of alarm call comprehension, usage and production in mangabeys occurs during juvenile stage, which may be simultaneously refined by observing other individuals and through individual experience. In a recent study, we found that mangabeys can acquire predator knowledge from alarm calls by one-trial social learning (León et al. 2022). Rapid individual learning and flexibility in alarm call usage have been also demonstrated in adult West African green monkeys when exposed to a novel threat, i.e., a remotely operated drone (Wegdell et al. 2019). Thus, innate knowledge seems unlikely and the appropriate categorization and response of con- and heterospecific alarm calls could occur through a combination of body maturation and, to a greater extent, social and individual learning.

We have shown that mangabeys’ competent alarm call behaviour towards con- and heterospecific signals arises during juvenile stage. However, we did not determinate the exact age at which infants start identifying the different con- and heterospecific alarm call types. This should be addressed in future comparative research to test which socioecology and cognitive features may shape species-specific learning rates. In a recent field experiment on immature chimpanzees, subjects consistently produced alarm calls in response to an unfamiliar but potentially hazardous object, i.e., a large spider model, starting only at 80 months old (Dezecache et al. 2019). The later development of adult-like responses to alarm calls in mangabeys that we found (at least 24 months old) is in between the ages that have been reported for other monkeys and prosimians (6–12 months old) (Seyfarth and Cheney 1980, 1986; Fischer et al. 2000; McCowan et al. 2001; Fichtel 2008) and chimpanzees. This suggests that further research on the ontogeny of alarm call comprehension in mangabeys could shed light to the cognitive division between apes and monkeys (Tomasello and Call 1994; Amici et al. 2010; Tomasello 2010)*.* Additionally, it would be interesting and informative to conduct an analysis of the acoustic features of the different mangabey alarm calls. Finally, due to the complexity of mangabeys’ snake-specific antipredator behaviour, this could be a promising model to explore the ability of primates to socially learn relevant contextual information related to their alarm calls.

In summary, this study provides insights on the developmental process by which young primates comprehend their own and other species’ alarm calls and display species-specific antipredator behaviours. Our findings illustrated how call comprehension starts eliciting simple but adaptative escape responses, as individuals simultaneously acquire more experience and receive inputs from other group members and heterospecifics. Eventually, escape responses diversify showing predator specificity. Our findings support the view of an oddly asymmetrical communication system in primates, wherein vocal comprehension, usage, and production exhibit fundamental differences in their flexibility and ontogeny, with vocal comprehension being highly flexible and preceding appropriate vocal usage and vocal production. Examining both conspecific and heterospecific information available to individuals during predator encounters is particularly valuable in shedding light on the development of alarm call behaviour, as primates inhabit ecosystems with multiple sources of information, including non-predatory heterospecifics. The acoustic variation of signals produced in these multi-information environments and additional contextual information, possibly together with learning mechanisms, allows listeners to select appropriate responses to their different predation pressures. While the degree to which natural selection favours social learning or alternative more general learning mechanisms to produce optimal anti-predatory behavioural strategies remains an open question, there is no doubt that the animals’ ability to understand the meaning of their own and other species’ alarm calls is, to a large extent, a learning process that occurs during their early stages of life and refine throughout their maturation process.

## Supplementary Information

Below is the link to the electronic supplementary material.

Supplementary file1 (DOCX 6716 KB)

Supplementary file2 (MP4 72306 KB)

Supplementary file3 (MP4 63169 KB)

Supplementary file4 (MP4 66536 KB)

Supplementary file5 (MP4 86159 KB)

## Data Availability

The datasets generated during and/or analysed during the current study are available in the OSF repository, https://osf.io/zrgdj/. All additional material related to this study, including video recordings and sound files, may be requested from the corresponding author.

## Code availability

R code is available at https://osf.io/zrgdj/.
