# Supplementary material for: Comprehension of own and other species’ alarm calls in sooty mangabey vocal development
Source: Behav Ecol Sociobiol. 2023 May 23;77(5):56. doi: 10.1007/s00265-023-03318-6 (PMC10205891; doi:10.1007/s00265-023-03318-6)
Supplement: Supplementary file 1 — Supplementary file1 (DOCX 6716 KB) [file 265_2023_3318_MOESM1_ESM.docx]

**Supplemental material**

Article title: **Comprehension of own and other species’ alarm calls in sooty mangabey vocal development**

Journal name: **Behavioral ecology and sociobiology**

Julián León^1, 2,*^, Constance Thiriau^1, 2^, Catherine Crockford^3,4^ and Klaus Zuberbühler^1, 2,5^

^1^Institute of Biology, University of Neuchâtel, 2000 Neuchâtel, Switzerland.

^2^Taï Monkey Project, Centre Suisse de Recherches Scientifiques, 01 BP1303 Abidjan 01, Cote d’Ivoire.

^3^Tai Chimpanzee Project, Centre Suisse de Recherches Scientifiques, 01 BP1303 Abidjan 01, Cote d’Ivoire.

^4^Institute of Cognitive Sciences Marc Jeannerod, CNRS, 69330, Lyon, France

^5^School of Psychology and Neuroscience, University of St. Andrews, KY16 9JP Saint Andrews, Scotland (UK).

^*^Corresponding author: julian.unine@unine.ch

**Fig. S1** Representative samples of conspecific stimuli: Sooty mangabey alarm calls produce in response to (a) leopards, (b) eagles and (c) snakes. Spectrograms were made using Raven and the following settings: 1024 FFT, Hamming window, 75% overlap, 22.05 kHz sampling frequency

**Fig. S2** Representative samples of heterospecific stimuli: male Diana monkey loud alarm calls produce in response to (a) leopards, (b) eagles and (c) falling trees. Spectrograms were made using Raven and the following settings: 1024 FFT, Hamming window, 75% overlap, 22.05 kHz sampling frequency

**Fig. S3** Responses to conspecific alarm calls: Proportion of individuals that showed appropriate specific antipredator behavioural responses to conspecific alarm call playbacks. Grey dots indicate individual values for each subjects’ response. Post hoc pairwise comparisons: * *P*<0.05

**Table S1** Number of trials of the different playback conditions for each age class

| **Conspecific trials** | | | |
| --- | --- | --- | --- |
| Age class  (number of subjects) | Adults  (N=10) | Old juveniles  (N=11) | Young juveniles  (N=10) |
| Playback stimulus | Number of trials | | |
| Leopard alarm | 4 | 7 | 6 |
| Eagle alarm | 6 | 5 | 5 |
| Snake alarm | 5 | 6 | 5 |
| **Heterospecific trials** | | | |
| Age class | Adults  (N=15) | Old juveniles  (N=13) | Young juveniles  (N=11) |
| Playback stimulus | Number of trials | | |
| Leopard alarm | 5 | 6 | 6 |
| Eagle alarm | 8 | 8 | 7 |
| Tree alarm | 6 | 9 | 8 |

Sooty mangabey and Diana monkey alarm calls rates.

To carry out our experiments with a frequency no higher than the natural one, from August to October 2019 (N=47 days), we took *ad libitum* data during all-day samplings on the natural rate of sooty mangabeys’ exposure to their own and Diana monkey different alarm call types. After an alarm call was registered, all following calls referring to the same threat were considered as part as one calling event and were not considered independent. We considered new calling events to occur only if no other predator-related vocalization was emitted by any primate species during the previous hour. JL and two field assistants (PK and LB) took the data with a perfect interrater reliability of Cohen’s kappa=1 for calling events classification (Table S1).

**Table S2** Natural occurrences of alarm calling events of sooty mangabeys (conspecifics) and Diana monkeys (heterospecifics)

|  | Alarm Call Type | | |
| --- | --- | --- | --- |
| *Conspecific alarm calls* | Leopard | Eagle | Snake |
| Alarm calling events | 7 | 60 | 19 |
| Calling events/week (±SE) | 1.04 (0.06) | 8.94 (0.19) | 2.83 (0.07) |
| *Heterospecific alarm calls* | Leopard | Eagle | Falling tree |
| Alarm calling events | 4 | 72 | 49 |
| Calling events/week (±SE) | 0.6 (0.04) | 10.72 (0.21) | 7.3 (0.2) |
